# Translation and cross cultural adaptation of the questionnaire “Quality of Alimentation” for brazilian portuguese

**DOI:** 10.1590/2317-1782/20242023168en

**Published:** 2024-05-31

**Authors:** Maria da Conceição Queiroz Lomachinsky, Suzana Lins da Silva, Cynthia Meira de Almeida Godoy, Flavio Augusto Martins Fernandes

**Affiliations:** 1 Programa de Pós-graduação stricto sensu Mestrado Profissional em Cuidados Intensivos do Instituto de Medicina Integral Prof. Fernando Figueira – IMIP – Recife (PE), Brasil.; 2 Departamento de Fonoaudiologia, Hospital Universitário Onofre Lopes, Universidade Federal do Rio Grande do Norte – UFRN – Natal (RN), Brasil.

**Keywords:** Food Intolerance, Bariatric Surgery, Indicators of Quality of Life, Surveys and Questionnaires, Translating, Cross-Cultural Comparison

## Abstract

**Purpose:**

We aimed to provide translation and cultural adaptation of the questionnaire “Quality of Alimentation” from English to Brazilian Portuguese.

**Methods:**

The transcultural translation process consisted of the following steps: translation of the original English version to Portuguese by two bilingual translators native in the targeted language; Reverse translation by two translators native in the original language; Review of reverse translation; Review of the Portuguese version from the questionnaire by a local committee of experts in bariatric surgery; Pre-trial to evaluate of clarity, comprehension, and overall acceptability by the target population.

**Results:**

In its final Portuguese version, the questionnaire “Quality of alimentation” was found to be of clear comprehension and easy applicability.

**Conclusion:**

The questionnaire’s translation and cultural adaptation for Brazilian Portuguese represents an important step towards improving food tolerance evaluation following bariatric surgery. Further studies are however necessary for validation of its psychometric properties in Brazil.

## INTRODUCTION

Obesity is a public health problem, now understood as a phenomenon with multiple causes that encompass genetic, behavioral, psychological, social, metabolic, and endocrine components^(1,2)^. Among the existing treatments for obesity, various interventions can be listed: behavioral, pharmacological, and, not infrequently, surgical^(3-5)^. In terms of surgical intervention, one of the main complications of gastroplasty is the development of food intolerance, with Brazilian studies reporting prevalence rates of up to 42.6%^(6)^.

This issue pertains to acceptance problems linked to specific groups of foods that may lead to regurgitation and/or vomiting. From a pathophysiological standpoint, food intolerance can be determined by multiple factors, ranging from inherent differences in surgical technique to the emergence of mechanical complications in the postoperative period^(7)^. Very often, this issue is exacerbated by inefficiency in chewing, a potentially preventable cause that can be rehabilitated^(8)^.

The impact of food intolerance is significant both socially and nutritionally. Socially, many patients avoid meals with friends and loved ones out of fear and embarrassment of symptoms. In addition, they often tend to replace solid consistency with mushy and semi-liquid consistencies, generally high in calories, a factor that contributes to weight gain in the medium and long term^(7-9)^.

Although already recognized in the literature by the medical communities of surgery and endocrinology, this phenomenon manifests itself through a spectrum of symptoms perceived subjectively by patients, making its measurement difficult and surrounded by stigma^(6,10,11)^.

The quantification of the degree of intolerance, however, has practical implications for treatment direction and also scientific implications for the refinement of techniques and categorization of surgical outcomes^(7)^. In this sense, for the standardized quantification of the degree of food intolerance, the “Quality of Alimentation” questionnaire by Suter et al.^(7)^ stands out for its simplicity and applicability to all types of surgical techniques. Its use by the multidisciplinary team can aid in the early detection of food intolerance, contributing to planning directed towards the patient's needs^(7,12)^.

To date, there are no publications on the validation of food tolerance screening questionnaires in the postoperative period of bariatric surgery in Brazil. Thus, the translation and adaptation of the questionnaire by Suter, Calmes, Paroz, and Giusti^(7)^. to Brazilian Portuguese is necessary, with the aim of optimizing the care of patients undergoing bariatric surgery and allowing the standardization of the measure of food intolerance in future research.

## METHODS

The present study is a methodological research on the translation and cross-cultural adaptation of the "Quality of Alimentation" questionnaire^(7)^. This study was initiated after receiving authorization from the original author, Dr. Michael Suter, from the Riviera-Chablais Hospital in Rennaz, Switzerland, requested via email and promptly accepted, following the approval of the ethics committee number 5,259,249.

The procedures adopted in this study for the translation followed the principles common to the models proposed by Reichenheim and Moraes^(13)^, Beaton et al.^(14)^, and Pernambuco et al.^(15)^ involving 6 steps: (1) direct translation to the target language (Portuguese); (2) synthesis of the translations; (3) back-translation to the original language (English); (4) coherence analysis; (5) analysis by a committee of judges experts in the topic; (6) pre-test in a target population; review and construction of the final version.

The professionals who composed the committee of judges and the adult participants involved in the research were informed about the purpose of the study and consented to participate through the Free and Informed Consent Term (TCLE), adhering to the ethical criteria of Resolution 466/12.

### Stage 1: Direct Translation

In this stage, two bilingual translators whose native language is Brazilian Portuguese conducted two direct and independent translations of the Original Version (VO) of the "Quality of Alimentation" questionnaire into Portuguese, resulting in two Portuguese-translated versions by translators 1 and 2, VPT 1 and VPT 2.

### Stage 2: Translation Synthesis

At this stage, a meeting was held with the translators and responsible researchers, where the two translations resulting from the previous step were delivered. Stage 2 was carried out with the help of a table containing all the items from the Original Version (VO) and the translated versions into Portuguese (VPT1 and VPT2), aiming to assess the linguistic, semantic, idiomatic, conceptual, and contextual discrepancies and obtain a unique version. In this stage, the translations were compared by the two translators and responsible researchers, differences between the translated versions were identified, and necessary adjustments were made until consensus was reached (VST).

### Stage 3: Back-Translation

During this stage, the synthesis version from the target language was back-translated into the original language by two independent bilingual translators (VIT1 and VIT2), whose native language matched the original questionnaire (English). They did not have access to the original questionnaire. After this process was completed, a meeting was held between the researchers and the translators involved in the back-translation to compare the two versions, discuss the discrepancies, and correct possible translation errors that could compromise meanings, leading to semantic and conceptual differences. This meeting resulted in a new synthesis version of the back-translation (VST2).

### Stage 4: Coherence Analysis

From there, a fifth bilingual translator, whose native language is Brazilian Portuguese and who was blind to the previous translations into Portuguese, translated the back-translation synthesis (VST2) into Brazilian Portuguese (VPT3) with the aim of not only evaluating the equivalence of the back-translated English versions with the original version but also assessing content coherence among the multiple translations.

### Stage 5: Consolidation by a Committee of Judges

A multidisciplinary group of professionals, experts, masters, or doctors with at least five years of experience in the area of bariatric surgery was included in the committee of judges. The committee involved: 03 surgeons, 03 speech therapists, 03 endocrinologists, and 03 nutritionists. Each professional received a virtual form where the Free and Informed Consent Term, the Participant Declaration, a Table with the Original Version (VO), Portuguese Translated Versions (VPT1 and VPT2), Translation Synthesis Version (VST), English Translated Versions (VIT1 and VIT2), and Portuguese Version of the Back-Translation Synthesis (VPT3) were attached. The form also included a brief explanation about the types of equivalences that the judges should consider, as proposed by Beaton et al.^(14)^ From there, each judge could select the alternative they deemed as the best and most equivalent translation for each questionnaire item. The discrepancies were discussed and analyzed by the principal researcher to select only one alternative for each item.

### Stage 6: Pre-Test

At this moment, the Adapted Version (VA) for the Portuguese language, resulting from the analysis by the committee of judges, was applied independently by the principal researcher and by a collaborating speech therapist to a random and consecutive sample of 40 patients divided equally between each professional. The interviews were conducted at different times by each interviewer. Initially, 20 questionnaires along with the scale were applied by the principal researcher, and at another time, 20 questionnaires and scales by the collaborating speech therapist. The eligibility criteria for participant selection were:

Follow-up in the outpatient bariatric surgery service of the Institute of Integral Medicine Prof. Fernando Figueira- IMIP in the pre-surgical or post-surgical period and Age between 18 to 65 years, which is considered the maximum age accepted by the bariatric surgery program. No exclusion criteria were applied based on socioeconomic or educational levels.

The pre-test aimed to evaluate the understanding and clarity of the culturally adapted and translated instrument. The 11 items of the questionnaire were evaluated through questions and answers, in a face-to-face interview format, conducted by the research speech therapist and the collaborating speech therapist, which required about 2 to 5 minutes for the evaluation of each participant. After completing the questionnaire, a colored illustrative Likert visual scale was presented to the interviewees to assess the ease of understanding and clarity of each questionnaire item. The guiding question for all items was "Was the question clear and easy to understand?", with five response options: 1-Strongly disagree; 2-Disagree somewhat; 3-Indifferent; 4-Agree somewhat; 5-Strongly agree.

This study did not aim to validate the Portuguese version of the questionnaire. Therefore, the food intolerance scores obtained for each patient were not communicated to the other team members, and the conduct of each professional remained unchanged.

## RESULTS

After the first stage of the translation, two versions of the questionnaire were obtained in Portuguese. In the synthesis of the versions, there were discrepancies in 08 items of the questionnaire (Chart 1). After discussion among the translators and the principal researcher, a combination of the versions was considered, as the translations were similar, and the different terms were synonyms.

**Chart 1:** Description of the translations and back-translations evaluated by the committee of judges and the principal author. The objective was universal equivalence with an emphasis on semantic and cultural spheres between the original version and the pre-final version

	Original Version	Portuguese translation version 1	Portuguese translation version 2	Translation Synthesis in Portuguese	English back-translation version 1	English back-translation version 2	S ynthesis version of the back-translation	Portuguese Version of the Back-Translation Synthesis	Equivalences
	**Quality of Alimentation**	**Qualidade da Alimentação**	**Qualidade da Alimentação**	**Qualidade da Alimentação**	**Nutritional Quality**	**Dietary Quality**	**Dietary Quality**	**Qualidade da Dieta**	
**1)**	**How would you rate your overall satisfaction regarding how you can eat presently?**	**Como você avalia sua satisfação em relação ao que você pode comer atualmente?**	**Como você classificaria sua satisfação em geral com a forma como você come atualmente?**	**Como você avalia sua satisfação em geral, com a forma como você come atualmente?**	**How would you rate your overall satisfaction with your current eating habits?**	**How do you assess your overall satisfaction with your current eating habits?**	**How would you rate your overall satisfaction with your current eating habits?**	**Como você classificaria sua satisfação geral com seus hábitos alimentares atuais?**	Semantic
	Excellent	Excelente	Excelente	Excelente	Excellent	Excellent	Excellent	Excelente	
	Good	Boa	Boa	Boa	Good	Good	Good	Bom	
	Acceptable	Aceitável	Aceitável	Aceitável	Fair	Acceptable	Acceptable	Aceitável	
	Poor	Ruim	Ruim	Ruim	Poor	Bad	Poor	Ruim	
	Very Poor	Muito Ruim	Muito Ruim	Muito Ruim	Very Poor	Very Bad	Very Poor	Muito ruim	
**2)**	**Why?**	**Por que?**	**Por quê?**	**Por quê?**	**Why?**	**Why?**	**Why?**	**Por que?**	Semantic
**3)**	**How many meals do you eat a day?**	**Quantas refeições por dia você faz?**	**Quantas refeições você faz por dia?**	**Quantas refeições você faz por dia?**	**How many meals do you eat per day?**	**How many meals do you have per day?**	**How many meals do you eat per day?**	**Quantas refeições você faz por dia?**	Semantic
**4)**	**Among the following meals, which one do you have?**	**Quais das seguintes refeições você faz?**	**Dentre as refeições a seguir, quais você faz?**	**Dentre as refeições a seguir, quais você faz?**	**Which of the following meals do you prepare yourself?**	**Which of the following meals do you eat?**	**Which of the following meals do you eat?**	**Quais das seguintes refeições você faz?**	Semantic
	Breakfast	Café da manhã	Café da manhã	Café da manhã	Breakfast	Breakfast	Breakfast	Café da manhã	
	Lunch	Almoço	Almoço	Almoço	Lunch	Lunch	Lunch	Almoço	
	Supper	Jantar	Jantar	Jantar	Dinner	Dinner	Dinner	Jantar	
**5)**	**Which of them constitutes your daily main meal?**	**Qual delas é a sua principal refeição do dia?**	**Qual destas é a sua refeição principal?**	**Qual delas é a sua principal refeição do dia?**	**Which of these meals is usually your largest meal?**	**Which one of them is your main daily meal?**	**Which one of them is your daily main meal?**	**Qual delas é a sua principal refeição diária?**	Semantic
**6)**	**Do you eat between meals?**	**Você come entre as refeições?**	**Você come entre as refeições?**	**Você come entre as refeições?**	**Do you snack in between meals?**	**Do you eat between meals?**	**Do you eat between meals?**	**Você come entre as refeições?**	Semantic
	Yes	Sim	Sim	Sim	Yes	Yes	Yes	Sim	
	No	Não	Não	Não	No	No	No	Não	
**7)**	**If yes, When?**	**Caso sua resposta seja sim, quando?**	**Se sim, quando?**	**Caso sua resposta seja sim, quando?**	**If yes, during what part of the day?**	**If you responded yes, When?**	**If yes, when?**	**Se sim, quando?**	Semantic
	Morning	Manhã	Manhã	Manhã	Morning	Morning	Morning	Manhã	
	Afternoon	Tarde	Tarde	Tarde	Afternoon	Afternoon	Afternoon	Tarde	
	Evening	Noite	Noite	Noite	At night	Evening	Evening	Noite	
**8)**	**Can you eat everything?**	**Você pode comer de tudo?**	**Você consegue comer tudo?**	**Você consegue comer de tudo?**	**Are you able to finish your meals?**	**Are you able to eat everything?**	**Are you able to eat all kinds of food?**	**Você consegue comer todos os tipos de alimentos?**	Semantic
	Yes	Sim	Sim	Sim	Yes	Yes	Yes	Sim	
	No	Não	Não	Não	No	No	No	Não	
**9)**	**More specifically, how can you eat?**	**Mais especificamente, como pode comer?**	**Mais especificamente, como você consegue comer?**	**Mais especificamente, como você consegue comer?**	**More specifically, please rate how easy you find it to eat?**	**Specifically, how are you able to eat?**	**Please rate how you find eating the following foods?**	**Por favor,**	Semantic
								**Avalie o que você acha de comer os seguintes alimentos?**	
	(Easily-With Some Difficulties-Not at all)	(Com facilidade-Com alguma dificuldade-De forma alguma)	(Facilmente-Com alguma dificuldade-Não consigo de jeito nenhum)	(Facilmente-Com alguma dificuldade-Não consigo de jeito nenhum)	(Easy-Somewhat difficult-Impossible)	(Easily-With some difficulty-Unable to eat at all)	(Easy-Somewhat difficult-Impossible)	(Fácil-Um pouco difícil-Impossível)	
	Red meat	Carne vermelha	Carne vermelha	Carne vermelha	Beef	Red meat	Red meat	Carne vermelha	
	White meat	Carne branca	Carne branca	Carne branca	White meat	White meat	White meat	Carne branca	
	Salad	Salada	Salada	Salada	Salad	Salad	Salad	Salada	
	Vegetables	Legumes	Vegetais	Legumes	Vegetables	Vegetables	Vegetables	Vegetais	
	Bread	Pão	Pão	Pão	Bread	Bread	Bread	Pão	
	Rice	Arroz	Arroz	Arroz	Rice	Rice	Rice	Arroz	
	Pasta	Massa	Massa	Massa	Pasta	Pasta	Pasta	Massa	
	Fish	Peixe	Peixe	Peixe	Fish	Fish	Fish	Peixe	
**10)**	**Are there other types of food that you cannot eat at all?**	**Há outros alimentos que você não pode comer de forma alguma?**	**Há outros tipos de comida que você não consegue comer de jeito nenhum?**	**Há outros tipos de alimentos que você não consegue comer de jeito nenhum?**	**Are there any other types of foods you are unable to eat?**	**What other types of food are you completely unable to eat?**	**Are there any other types of foods you are unable to eat at all?**	**Existem outros tipos de alimentos**	Semantic
								**que você não consegue comer?**	
**11)**	**Do you vomit/**	**Você vomita/**	**Você vomita/**	**Você vomita/**	**Do you vomit/**	**Do you vomit/**	**Do you vomit/**	**Vomita/**	Semantic/Grammatical/Semantic
	**regurgitate?**	**regurgita?**	**regurgita?**	**regurgita?**	**throw up?**	**regurgitate?**	**regurgitate?**	**regurgita?**	
	Daily	Diariamente	Diariamente	Diariamente	Daily	Daily	Daily	Diário	
	Often (>2x/week)	Com frequência (> duas vezes por semana)	Com frequência (>2x por semana)	Com frequência (maior que 2 vezes por semana)	Often (more than twice a week)	Frequently (more than 2 times per week)	Often (more than twice a week)	Frequentemente (mais de duas vezes por semana)	
	Rarely	Raramente	Raramente	Raramente	Rarely	Rarely	Rarely	Raramente	
	Never	Nunca	Nunca	Nunca	Never	Never	Never	Nunca	

In the back-translation of the Portuguese version to English, two versions were also obtained. In the synthesis of the versions, there were discrepancies in 10 items. Again, after discussion between both translators involved and the principal researcher, a combination of the versions was considered, as the translations were similar, and the different terms were synonyms.

The synthesis of the translated and back-translated versions was consensually performed by each pair of involved translators, comparing it in the end with the original version to assess semantic, idiomatic, conceptual, linguistic, and contextual discrepancies.

During the evaluation process by the committee of judges, there was a majority of responses sent (items 02, 03, 04, 05, 06, 07, 08, 10) agreeing and preferring the semantic equivalence of the alternatives present in version VPT3. These items were, therefore, fully incorporated into the Adapted Version (VA) for the pre-test phase.

Furthermore, the following adjustments were necessary: for Item 1 of the original version “How would you rate your overall satisfaction regarding how you can eat presently?" one of the judges found it better translated in the Portuguese translation version 2 (VPT2) as “Como você classificaria sua satisfação em geral com a forma como você come atualmente?" and another judge judged it better translated in the Portuguese translation version 1 (VPT1) as “Como você avalia sua satisfação em relação ao que você pode comer atualmente?" Having the other judges opted for “Como você classificaria sua satisfação geral com seus hábitos alimentares atuais?" as shown in VPT3. Due to greater agreement among the judges, this version was chosen.

For item 9 of the original version “More specifically, how can you eat?" one of the judges also found it better translated in the Portuguese translation version 2 (VPT2) as “Mais especificamente como você consegue comer?" Having the other judges opted for "Por favor, avalie o que você acha de comer os seguintes alimentos?" as shown in VPT3. Due to greater agreement among the judges, this version was chosen.

For Item 11 of the original version “Do you vomit/regurgitate?" the response “Daily” which in version VPT3 was translated as “diário” in the Adapted Version (VA) was changed to “diariamente” based on the suggestion of 02 judges to preserve the agreement with “frequentemente” and “raramente”. Also in item 11, it was necessary to add the pronoun “você” in the question “Você vomita/regurgita?" to preserve parallelism with the other items in the questionnaire.

In the pre-test phase, all 11 items of the Adapted Version (VA) of the questionnaire (Appendix 1) were evaluated as clear and easy to understand (grade 5 on the Likert Scale) by the patients who underwent the application, of which 30 were in the pre-operative period and 10 in the post-operative period.

## DISCUSSION

Until the publication of the "Quality of Alimentation" questionnaire by Suter, Calmes, Paroz, and Giusti^(7)^, there were no other tools available for quantifying food intolerance in patients undergoing bariatric surgery. As pointed out in the recent Brazilian systematic review by Stumpf et al.^(11)^, which analyzed the measurement of food tolerance through this instrument, the differential lies in the ability to quantify the subjectivity of the complaint through the score^(11)^. The questionnaire is simple and has been used internationally to describe the evolution of food tolerance in the short, medium, and long term, proving to be consistent across many cultures^(11,16-18)^.

Although there is no methodological consensus considered the gold standard for translation and cross-cultural adaptation, this study strictly adhered to the main steps recommended by the literature: translation and back-translation, review by a committee of expert judges, and pre-testing in the target population^(14,19,20)^.

The term "transcultural" emphasizes a process that considers both the language and culture of the population in question, in order to maintain equivalence of meaning. The assumption of conceptual equivalence that allowed us to initiate the translation process stems from the observation of the universality of the symptoms addressed, which are reported by patients from multiple cultures and appear to be intrinsic to the restrictive nature of surgical procedures^(6,7,11)^.

As proposed by Herdman et al. and followed in multiple national and international translation protocols, transcultural adaptation from a universalist perspective is necessary, beyond direct and grammatical parity between terms, other equivalences^(14,15,20,21)^.

Given the simple and straightforward nature of this questionnaire, this task did not prove to be problematic. This is evidenced by the great agreement in the analysis by the committee of judges and the coherence between the multiple translated and back-translated versions.

Eight of 11 items were able to be incorporated into the final version without any necessary changes in relation to version VPT3 with full agreement from all the involved judges. The discrepancies in the remaining items dealt with aesthetic issues and were easily overcome after discussion with the involved Judges. The only change that was necessary after the analysis was restricted to item (item 11) and aimed to ensure grammatical parallelism, having been applied without opposition from any professional.

It is hypothesized that the great success found in this process is due not only to the cultural proximity between the countries that have been adopting the questionnaire but also to the simplicity of the instrument, which consists of only 11 questions and addresses in a practical and objective manner symptoms similarly described by patients around the world.

Despite the success found in the pre-test phase and the inclusion of patients from a diverse profile in a large public health service located at the Institute of Integral Medicine Prof. Fernando Figueira- IMIP, it is emphasized that this work did not propose to validate the translated questionnaire. Here is presented a methodological study, focused on bridging the social and cultural distances that might hinder the use of the instrument by Suter et al.^(7)^ for the Brazilian population.

As a limitation of our work, we emphasize that due to the unavailability in the Brazilian literature of other studies on the translation and cross-cultural adaptation of food tolerance questionnaires, it was not possible to perform a comparison of our results with previous studies.

## CONCLUSION

This study facilitated the translation and cross-cultural adaptation of the "Quality of Alimentation" instrument from English to Brazilian Portuguese, making it ready for the next steps in the validation process.

## Appendix 1 "Quality of Diet" translated and cross-culturally adapted to Brazilian Portuguese

QUALIDADE DA DIETA

Nome: __________________________________

Sobrenome: __________________________________

Número de meses após a cirurgia: _______ meses.

Como você classificaria sua satisfação geral com seus hábitos alimentares atuais?

**Table:**

Excelente	
Bom	
Aceitável	
Ruim	
Muito ruim	

Por que? __________________________________

Quantas refeições você faz por dia? __________________________________

Qual das seguintes refeições você faz?

**Table:**

Café da manhã	
Almoço	
Jantar	

Qual delas é a sua principal refeição diária? _____________________________

Você come entre as refeições?

**Table:**

Sim	
Não	

Se sim, quando?

**Table:**

Manhã	
Tarde	
Noite	

Você consegue comer todos os tipos de alimentos?

**Table:**

Sim	
Não	

Por favor, avalie o que você acha de comer os seguintes alimentos?

**Table:**

	Fácil	Um pouco difícil	Impossível
Carne vermelha			
Carne branca			
Salada			
Vegetais			
Pão			
Arroz			
Massa			
Peixe			

Existem outros tipos de alimentos que você não consegue comer?

________________________________________________________________

Você vomita/regurgita?

**Table:**

Diariamente	
Frequentemente (mais de duas vezes por semana)	
Raramente	
Nunca	
