# Supplementary material for: Translation and cross cultural adaptation of the questionnaire “Quality of Alimentation” for brazilian portuguese
Source: Codas. 2024 May 31;36(4):e20230168. doi: 10.1590/2317-1782/20242023168en (PMC11189149; doi:10.1590/2317-1782/20242023168en)
Supplement: Translation

# Translation: Tradução e adaptação transcultural do questionário “Quality of Alimentation” para o português brasileiro

**DOI:** 10.1590/2317-1782/20242023168pt

**Authors:** Maria da Conceição Queiroz Lomachinsky, Suzana Lins da Silva, Cynthia Meira de Almeida Godoy, Flavio Augusto Martins Fernandes

**Affiliations:** 1 Programa de Pós-graduação stricto sensu Mestrado Profissional em Cuidados Intensivos do Instituto de Medicina Integral Prof. Fernando Figueira – IMIP – Recife (PE), Brasil.; 2 Departamento de Fonoaudiologia, Hospital Universitário Onofre Lopes, Universidade Federal do Rio Grande do Norte – UFRN – Natal (RN), Brasil.

**Keywords:** Intolerância Alimentar, Cirurgia Bariátrica, Indicadores de Qualidade de vida, Inquéritos e Questionário, Tradução, Comparação Transcultural

## Abstract

**Objetivo:**

Traduzir e adaptar transculturalmente o questionário “Quality of Alimentation” do inglês para a língua portuguesa do Brasil.

**Método:**

O processo de tradução e adaptação transcultural do questionário “Quality of Alimentation” segue as seguintes etapas: tradução por dois tradutores bilíngues nativos do idioma alvo, síntese das versões e retradução por dois tradutores nativos do idioma de origem e, por fim, revisão da retradução para submissão a um comitê de juízes especialistas. Uma vez aprovado, o questionário seguiu para teste com usuários a fim de avaliar a clareza, compreensibilidade e aceitabilidade da versão traduzida.

**Resultados:**

Na versão final em português brasileiro do questionário “Quality of Alimentation” o instrumento mostrou-se de claro entendimento e fácil aplicabilidade.

**Conclusão:**

O questionário traduzido e adaptado para o português brasileiro, representa um passo significativo para melhora na avaliação da intolerância alimentar pós cirurgia bariátrica. Novos estudos são necessários para a validação das propriedades psicométricas do instrumento no Brasil.

## INTRODUÇÃO

A obesidade é um problema de saúde pública, atualmente entendida como um fenômeno de múltiplas causas que engloba componentes genéticos, comportamentais, psicológicos, sociais, metabólicos e endócrinos^(1,2)^. Dentre os tratamentos existentes para obesidade, podem-se listar diversas intervenções: comportamentais, medicamentosas e, não infrequentemente, cirúrgicas^(3-5)^.

Em relação à intervenção cirúrgica, uma das principais complicações da gastroplastia é o desenvolvimento de intolerância alimentar, com estudos brasileiros reportando valores de até 42.6% de prevalência^(6)^.

Trata-se de um problema de aceitação atrelado a grupos específicos de alimentos que pode evoluir com regurgitação e/ou vômitos. De um ponto de vista patofisiológico, a intolerância alimentar pode ser determinada por múltiplos fatores, desde diferenças inerentes a técnica cirúrgica, ao surgimento de complicações mecânicas no período pós-operatório^(7)^. Muito comumente, essa problemática é acentuada pela ineficiência na mastigação, causa potencialmente prevenível e passível de reabilitação^(8)^.

O impacto da intolerância alimentar faz-se tanto na esfera social como na nutricional. Socialmente, muitos pacientes evitam refeições com amigos e entes queridos por medo e vergonha dos sintomas. Além disso, frequentemente, exibem a tendência de substituir a consistência sólida para pastosa e semilíquida, em geral com alto teor de calorias, fato que contribui para o ganho de peso a médio e longo prazo^(7-9)^.

Apesar de já reconhecido em literatura pelas comunidades médicas de cirurgia e de endocrinologia, esse fenômeno manifesta-se através de um espectro de sintomas percebidos de forma subjetiva pelos pacientes o que torna sua mensuração difícil e cercada de estigma^(6,10,11)^.

A quantificação do grau de intolerância, entretanto tem implicações práticas para direcionamento de tratamento e também implicações científicas para aprimoramento de técnicas e categorização de desfechos cirúrgicos^(7)^.

Nesse sentido, na quantificação padronizada do grau de intolerância alimentar, o questionário “*Quality of Alimentation*” de Suter et al.^(7)^ se sobressai pela simplicidade e aplicabilidade a todos os tipos de técnicas cirúrgicas. Seu emprego pela equipe multidisciplinar pode auxiliar na detecção precoce da intolerância alimentar contribuindo para um planejamento direcionado às necessidades do paciente^(7,12)^.

Não existem até o momento, publicações acerca da validação de questionários de rastreamento de tolerância alimentar no pós operatório de cirurgia bariátrica no Brasil. Assim, a tradução e adaptação do questionário de Suter et al.^(7)^ para o português brasileiro faz-se necessária, com o intuito de otimizar a assistência aos pacientes submetidos à cirurgia bariátrica e permitir a padronização da medida de intolerância alimentar em pesquisas futuras.

## MÉTODO

O presente estudo é uma pesquisa metodológica de tradução e adaptação transcultural do questionário “*Quality of Alimentation*”^(7)^. Esse estudo foi iniciado após autorização do autor original, Dr. Michael Suter, do Hospital Riviera-Chablais,em Rennaz, Suíça, solicitada e prontamente aceita, através de correio eletrônico e após a aprovação do comitê de ética número 5.259.249.

Os procedimentos adotados neste estudo para a tradução seguiram os princípios comuns aos modelos propostos por Reichenheim e Moraes^(13)^, Beaton et al.^(14)^ e Pernambuco et al.^(15)^ e envolveram as 6 etapas: (1) tradução direta para o idioma alvo (português); (2) síntese das traduções; (3) tradução-reversa para o idioma original (inglês); (4) Análise de coerência e (5) análise por comitê de juízes especialistas no tópico; (6) pré-teste em uma população-alvo; revisão e construção da versão final.

Os profissionais que compuseram o comitê de juízes e os participantes adultos envolvidos na pesquisa foram esclarecidos quanto à finalidade do estudo, e consentiram com a participação através do Termo de Consentimento Livre e Esclarecido (TCLE) obedecendo aos critérios éticos da Resolução 466/12.

### Etapa 1: Tradução direta

Nesta etapa, dois tradutores bilíngues cuja língua materna é o Português falado no Brasil, realizaram duas traduções diretas e independentes da Versão Original (VO) do questionário “*Quality of Alimentation*” para o Português, resultando em duas versões traduzidas para o Português pelos tradutores 1 e 2, VPT 1 e VPT 2.

### Etapa 2: Síntese das traduções

Nessa etapa, foi realizada uma reunião com os tradutores e pesquisadores responsáveis, onde foram entregues as duas traduções resultantes da etapa anterior. A etapa 2 foi realizada com o auxílio de uma tabela contendo todos os itens da Versão Original (VO) e das versões traduzidas para a língua portuguesa (VPT1 e VPT2), com o objetivo de avaliar as discrepâncias linguísticas, semânticas, idiomáticas, conceituais e contextuais e obter uma versão única. Nessa etapa, as traduções foram comparadas pelos dois tradutores e pesquisadores responsáveis e identificadas as diferenças entre as versões traduzidas e realizadas as adaptações necessárias até alcançar o consenso (VST).

### Etapa 3: Retradução

Durante essa etapa foi realizada a retradução da versão da síntese do idioma alvo para o idioma original por dois tradutores bilíngues (VIT1 e VIT2), independentes, e que apresentavam língua materna equivalente ao questionário original (inglês). Os mesmos não tiveram acesso ao questionário original. Depois de finalizado esse processo, foi realizada uma reunião entre os pesquisadores e os tradutores envolvidos na retradução com a finalidade de comparar as duas versões, discutir as divergências e corrigir os possíveis erros existentes de tradução que podiam comprometer os significados gerando diferenças semânticas e conceituais. Desta reunião resultou uma nova versão síntese da retradução (VST2).

### Etapa 4: Análise de Coerência

A partir daí foi realizada, por um quinto tradutor bilíngue, cuja língua materna é o Português falado no Brasil, cego as traduções anteriores para o português, a tradução da síntese da retraduçãoƒ (VST2) para o português brasileiro (VPT3) com o objetivo de permitir não apenas a avaliação de equivalência das versões inglesas retraduzidas com a versão original, mas também, de avaliar a coerência de conteúdo entre as múltiplas traduções.

### Etapa 5: Consolidação por um Comitê de Juízes

Foram incluídos no comitê de juízes um grupo multidisciplinar de profissionais especialistas, mestres ou doutores com vivência de pelo menos cinco anos na área de cirurgia bariátrica. O comitê envolveu: 03 cirurgiões, 03 fonoaudiólogos, 03 endocrinologistas e 03 nutricionistas.

Cada profissional recebeu um formulário virtual onde encontravam-se anexados o Termo de Consentimento Livre e Esclarecido, a Declaração do Participante, uma Tabela com a Versão Original (VO), Versões Traduzidas para a Língua Portuguesa (VPT1 e VPT2), Versão Síntese das Traduções (VST), Versões Traduzidas para Língua Inglesa (VIT1 e VIT2) e Versão em Português da Síntese da retradução (VPT3). O formulário incluiu também uma breve explicação sobre os tipos de equivalências que mereciam consideração dos juízes, conforme proposto por Beaton et al.^(14)^ A partir daí cada juiz poderia selecionar a alternativa que julgou como melhor e mais equivalente tradução para cada item do questionário. As divergências foram discutidas e analisadas pela pesquisadora principal de modo a selecionar apenas uma alternativa para cada item.

### Etapa 6: Pré-teste

Neste momento, a Versão Adaptada (VA) para língua portuguesa, resultante da análise pelo comitê de juízes, foi aplicada, de forma independente, pela pesquisadora principal e por um colaborador fonoaudiólogo em uma amostra aleatória e consecutiva de 40 pacientes divididos igualmente entre cada profissional. As entrevistas foram realizadas em momentos distintos por cada entrevistador. No primeiro momento foram aplicados 20 questionários acompanhados da escala pelo pesquisador principal e em outro momento 20 questionários e escala pelo fonoaudiólogo colaborador. Os critérios de elegibilidade para a seleção de participantes foram: Acompanhamento no serviço ambulatorial de cirurgia bariátrica do Instituto de Medicina Integral Prof. Fernando Figueira — IMIP no período pré-cirúrgico ou pós-cirúrgico e idade entre 18 até 65 anos que é considerada idade máxima aceita pelo programa de cirurgia bariátrica. Não foram aplicados critérios de exclusão baseados em níveis socioeconômicos ou educacionais.

O objetivo do pré-teste foi avaliar a compreensão e clareza do instrumento traduzido e adaptado culturalmente. Os 11 itens do questionário foram avaliados através de perguntas e respostas, tipo entrevista face a face, conduzidas pela fonoaudióloga pesquisadora e fonoaudiólogo colaborador, o que demandou cerca de 2 a 5 minutos para a avaliação de cada participante. Após a finalização do questionário foi apresentada a escala visual ilustrativa colorida de Likert aos entrevistados para que avaliassem a facilidade de compreensão e clareza de cada item do questionário. A pergunta norteadora para todos os itens era “A questão foi clara e de fácil compreensão?”, sendo cinco opções de respostas: 1-Discordo totalmente; 2-Discordo parcialmente; 3-Indiferente; 4-Concordo parcialmente; 5-Concordo totalmente.

Este estudo não objetivou realizar a validação da versão portuguesa do questionário. Sendo assim, os escores de intolerância alimentar obtidos para cada paciente não foram comunicados aos demais membros da equipe e a conduta de cada profissional permaneceu inalterada.

## RESULTADOS

Após a primeira etapa da tradução, foram obtidas duas versões do questionário na língua portuguesa. Na síntese das versões, houveram divergências em 08 itens do questionário (Quadro 1). Após discussão entre os tradutores e a pesquisadora principal, considerou-se uma combinação das versões, pois as traduções eram semelhantes, e os termos diferentes eram sinônimos.

**Quadro 1:** Descrição das traduções e retraduções avaliadas pelo comitê de juízes e pela autora principal. Objetivada equivalência universal com ênfase em esferas semânticas e culturais entre versão original e versão pré-final

	Versão original	Versão traduzida para o português 1	Versão traduzida para o português 2	Versão sintetizada em português	Versão traduzida para o inglês 1	Versão traduzida para o inglês 2	Versão sintetizada em inglês	Versão traduzida da síntese da retradução (VPT 3)	Equivalências
	**Quality of Alimentation**	**Qualidade da Alimentação**	**Qualidade da Alimentação**	**Qualidade da Alimentação**	**Nutritional Quality**	**Dietary Quality**	**Dietary Quality**	**Qualidade da Dieta**	
**1)**	**How would you rate your overall satisfaction regarding how you can eat presently?**	**Como você avalia sua satisfação em relação ao que você pode comer atualmente?**	**Como você classificaria sua satisfação em geral com a forma como você come atualmente?**	**Como você avalia sua satisfação em geral, com a forma como você come atualmente?**	**How would you rate your overall satisfaction with your current eating habits?**	**How do you assess your overall satisfaction with your current eating habits?**	**How would you rate your overall satisfaction with your current eating habits?**	**Como você classificaria sua satisfação geral com seus hábitos alimentares atuais?**	Semântica
	Excellent	Excelente	Excelente	Excelente	Excellent	Excellent	Excellent	Excelente	
	Good	Boa	Boa	Boa	Good	Good	Good	Bom	
	Acceptable	Aceitável	Aceitável	Aceitável	Fair	Acceptable	Acceptable	Aceitável	
	Poor	Ruim	Ruim	Ruim	Poor	Bad	Poor	Ruim	
	Very Poor	Muito Ruim	Muito Ruim	Muito Ruim	Very Poor	Very Bad	Very Poor	Muito ruim	
**2)**	**Why?**	**Por que?**	**Por quê?**	**Por quê?**	**Why?**	**Why?**	**Why?**	**Por que?**	Semântica
**3)**	**How many meals do you eat a day?**	**Quantas refeições por dia você faz?**	**Quantas refeições você faz por dia?**	**Quantas refeições você faz por dia?**	**How many meals do you eat per day?**	**How many meals do you have per day?**	**How many meals do you eat per day?**	**Quantas refeições você faz por dia?**	Semântica
**4)**	**Among the following meals, which one do you have?**	**Quais das seguintes refeições você faz?**	**Dentre as refeições a seguir, quais você faz?**	**Dentre as refeições a seguir, quais você faz?**	**Which of the following meals do you prepare yourself?**	**Which of the following meals do you eat?**	**Which of the following meals do you eat?**	**Quais das seguintes refeições você faz?**	Semântica
	Breakfast	Café da manhã	Café da manhã	Café da manhã	Breakfast	Breakfast	Breakfast	Café da manhã	
	Lunch	Almoço	Almoço	Almoço	Lunch	Lunch	Lunch	Almoço	
	Supper	Jantar	Jantar	Jantar	Dinner	Dinner	Dinner	Jantar	
**5)**	**Which of them constitutes your daily main meal?**	**Qual delas é a sua principal refeição do dia?**	**Qual destas é a sua refeição principal?**	**Qual delas é a sua principal refeição do dia?**	**Which of these meals is usually your largest meal?**	**Which one of them is your main daily meal?**	**Which one of them is your daily main meal?**	**Qual delas é a sua principal refeição diária?**	Semântica
**6)**	**Do you eat between meals?**	**Você come entre as refeições?**	**Você come entre as refeições?**	**Você come entre as refeições?**	**Do you snack in between meals?**	**Do you eat between meals?**	**Do you eat between meals?**	**Você come entre as refeições?**	Semântica
	Yes	Sim	Sim	Sim	Yes	Yes	Yes	Sim	
	No	Não	Não	Não	No	No	No	Não	
**7)**	**If yes, When?**	**Caso sua resposta seja sim, quando?**	**Se sim, quando?**	**Caso sua resposta seja sim, quando?**	**If yes, during what part of the day?**	**If you responded yes, When?**	**If yes, when?**	**Se sim, quando?**	Semântica
	Morning	Manhã	Manhã	Manhã	Morning	Morning	Morning	Manhã	
	Afternoon	Tarde	Tarde	Tarde	Afternoon	Afternoon	Afternoon	Tarde	
	Evening	Noite	Noite	Noite	At night	Evening	Evening	Noite	
**8)**	**Can you eat everything?**	**Você pode comer de tudo?**	**Você consegue comer tudo?**	**Você consegue comer de tudo?**	**Are you able to finish your meals?**	**Are you able to eat everything?**	**Are you able to eat all kinds of food?**	**Você consegue comer todos os tipos de alimentos?**	Semântica
	Yes	Sim	Sim	Sim	Yes	Yes	Yes	Sim	
	No	Não	Não	Não	No	No	No	Não	
**9)**	**More specifically, how can you eat?**	**Mais especificamente, como pode comer?**	**Mais especificamente, como você consegue comer?**	**Mais especificamente, como você consegue comer?**	**More specifically, please rate how easy you find it to eat?**	**Specifically, how are you able to eat?**	**Please rate how you find eating the following foods?**	**Por favor,**	Semântica
								**Avalie o que você acha de comer os seguintes alimentos?**	
	(Easily-With Some Difficulties-Not at all)	(Com facilidade-Com alguma dificuldade-De forma alguma)	(Facilmente-Com alguma dificuldade-Não consigo de jeito nenhum)	(Facilmente-Com alguma dificuldade-Não consigo de jeito nenhum)	(Easy-Somewhat difficult-Impossible)	(Easily-With some difficulty-Unable to eat at all)	(Easy-Somewhat difficult-Impossible)	(Fácil-Um pouco difícil-Impossível)	
	Red meat	Carne vermelha	Carne vermelha	Carne vermelha	Beef	Red meat	Red meat	Carne vermelha	
	White meat	Carne branca	Carne branca	Carne branca	White meat	White meat	White meat	Carne branca	
	Salad	Salada	Salada	Salada	Salad	Salad	Salad	Salada	
	Vegetables	Legumes	Vegetais	Legumes	Vegetables	Vegetables	Vegetables	Vegetais	
	Bread	Pão	Pão	Pão	Bread	Bread	Bread	Pão	
	Rice	Arroz	Arroz	Arroz	Rice	Rice	Rice	Arroz	
	Pasta	Massa	Massa	Massa	Pasta	Pasta	Pasta	Massa	
	Fish	Peixe	Peixe	Peixe	Fish	Fish	Fish	Peixe	
**10)**	**Are there other types of food that you cannot eat at all?**	**Há outros alimentos que você não pode comer de forma alguma?**	**Há outros tipos de comida que você não consegue comer de jeito nenhum?**	**Há outros tipos de alimentos que você não consegue comer de jeito nenhum?**	**Are there any other types of foods you are unable to eat?**	**What other types of food are you completely unable to eat?**	**Are there any other types of foods you are unable to eat at all?**	**Existem outros tipos de alimentos**	Semântica
								**que você não consegue comer?**	
**11)**	**Do you vomit/**	**Você vomita/**	**Você vomita/**	**Você vomita/**	**Do you vomit/**	**Do you vomit/**	**Do you vomit/**	**Vomita/**	Sintática/ Gramatical
	**regurgitate?**	**regurgita?**	**regurgita?**	**regurgita?**	**throw up?**	**regurgitate?**	**regurgitate?**	**regurgita?**	Semântica
	Daily	Diariamente	Diariamente	Diariamente	Daily	Daily	Daily	Diário	
	Often (>2x/week)	Com frequência (> duas vezes por semana)	Com frequência (>2x por semana)	Com frequência (maior que 2 vezes por semana)	Often (more than twice a week)	Frequently (more than 2 times per week)	Often (more than twice a week)	Frequentemente (mais de duas vezes por semana)	
	Rarely	Raramente	Raramente	Raramente	Rarely	Rarely	Rarely	Raramente	
	Never	Nunca	Nunca	Nunca	Never	Never	Never	Nunca	

Na retradução da versão em português para língua inglesa, também foram obtidas duas versões. Na síntese das versões, houve divergências em 10 itens. Novamente, após discussão entre ambos os tradutores envolvidos e a pesquisadora principal, considerou-se uma combinação das versões, pois as traduções eram semelhantes, e os termos diferentes eram sinônimos.

A síntese das versões traduzidas e retraduzidas foi realizada de forma consensual por cada dupla de tradutores envolvidos comparando-se ao fim com a versão original para avaliação das discrepâncias semânticas, idiomáticas, conceituais, linguísticas e contextuais.

Durante o processo de avaliação do comitê de juízes, houve na grande maioria das respostas enviadas (itens 02, 03 ,04, 05, 06, 07, 08, 10) concordância e preferência quanto à equivalência semântica das alternativas presentes na versão VPT3. Esses itens foram, portanto, incorporados integralmente à Versão Adaptada (VA) para a fase pré-teste.

Ademais, foram necessários os seguintes ajustes: no Item 1 da versão original “How would you rate your overall satisfaction regarding how you can eat presently? Para um dos juízes estava mais bem traduzido na versão 2 da tradução para português (VPT2) “Como você classificaria sua satisfação em geral com a forma como você come atualmente?” e outro juiz julgou que estava mais bem traduzido na versão 1 da tradução para o português (VPT1) “Como você avalia sua satisfação em relação ao que você pode comer atualmente? Tendo os demais juízes optado por “Como você classificaria sua satisfação geral com seus hábitos alimentares atuais?” conforme exposto em VPT 3. Devido à maior concordância entre os juízes, optou-se pela escolha dessa versão.

No item 9 da versão original “More specifically, how can you eat?” Para um dos juízes também estava melhor traduzido na versão 2 da tradução para o português (VPT 2) “Mais especificamente como você consegue comer?" Tendo os demais juízes optado por "Por favor, avalie o que você acha de comer os seguintes alimentos?” conforme exposto em VPT 3. Devido a maior concordância entre os juízes, optou-se pela escolha dessa versão.

No Item 11 da versão original “*Do you vomit/regurgitate*?” A resposta “*Daily*” que na versão VPT 3 foi traduzida como “diário” na Versão Adaptada (VA) conforme sugestão de 02 juízes foi substituída por “diariamente” para preservar a concordância com “frequentemente” e “raramente”. Também no item 11, foi necessário o acréscimo do pronome de tratamento “você” na pergunta “Você vomita/regurgita?” de modo a preservar o paralelismo com os outros itens do questionário.

Na fase de pré-teste, todos os 11 itens da Versão Adaptada (VA) do questionário (Apêndice 1) foram avaliados como claros e de fácil compreensão (grau 5 da Escala Likert) pelos pacientes que se submeteram à aplicação, dos quais 30 estavam no período pré-operatório e 10 no pós-operatório.

## DISCUSSÃO

Até a publicação do questionário de qualidade alimentar de Suter, Calmes, Paroz e Giusti^(7)^, não haviam outros instrumentos disponíveis para a quantificação da intolerância alimentar em pacientes submetidos a cirurgia bariátrica. Conforme apontado na recente revisão sistemática brasileira por Stumpf et al.^(11)^, que analisou a mensuração da tolerância alimentar através desse instrumento, o diferencial está no poder de quantificar a subjetividade da queixa através do escore^(11)^. O questionário é simples e, internacionalmente, já vem sendo utilizado para descrever a evolução da tolerância alimentar em curto, médio e longo prazo, mostrando-se consistente dentre muitas culturas^(11,16-18)^.

Embora não exista um consenso metodológico considerado padrão ouro para tradução e adaptação transcultural, este estudo obedeceu rigorosamente às principais etapas recomendadas pela literatura: tradução e retradução, revisão por um comitê de juízes especialistas e pré-teste na população alvo^(14,19,20)^.

O termo transcultural enfatiza um processo que considera tanto a linguagem como a cultura da população em questão, de modo a manter a equivalência de significado. O pressuposto de equivalência conceitual que nos permitiu iniciar o processo de tradução parte da constatação de universalidade dos sintomas abordados, que são relatados por pacientes de múltiplas culturas e aparentam ser intrínsecos à natureza restritiva dos procedimentos cirúrgicos^(6,7,11)^.

Conforme proposto por Herdman et al e seguido em múltiplos protocolos nacionais e internacionais de tradução, a adaptação transcultural a partir de uma perspectiva universalista considera necessário além da paridade direta e gramatical entre termos, as demais equivalências^(14,15,20,21)^.

Dada a natureza simples e direta deste questionário, essa tarefa não se mostrou problemática. Isso se evidencia pela grande concordância na análise do comitê de juízes e pela coerência entre as múltiplas versões traduzidas e retraduzidas.

Oito de 11 itens puderam ser incorporados à versão final sem qualquer alteração necessária em relação à versão VPT3 com integral concordância de todos os juízes envolvidos. As divergências nos demais itens trataram de questões estéticas e foram facilmente superadas após discussão com os Juízes envolvidos. A única alteração que se fez necessária após a análise restringiu-se ao item (item 11) e visou garantir o paralelismo gramatical, tendo sido aplicada sem oposição de nenhum profissional.

Hipotetiza-se que o grande sucesso encontrado neste processo se deve não apenas a proximidade cultural entre os países que vem adotando o questionário, mas também a simplicidade do instrumento, que consiste de apenas 11 perguntas e que aborda de forma prática e objetiva sintomas descritos similarmente por pacientes de todo o mundo.

Apesar do sucesso encontrado na fase pré-teste e da inclusão de pacientes de um perfil diverso em um grande serviço público de saúde localizado no Instituto de Medicina Integral Prof. Fernando Figueira — IMIP, ressalta-se que o presente trabalho não se propôs a validar o questionário traduzido. Aqui apresenta-se um estudo metodológico, focado em abreviar as distâncias sociais e culturais que porventura pudessem dificultar o uso do instrumento de Suter et al.^(7)^ para a população brasileira.

Como limitação do nosso trabalho ressaltamos que devido a indisponibilidade na literatura brasileira de outros estudos de tradução e adaptação transcultural de questionários de tolerância alimentar, não foi possível realizar comparação dos nossos resultados com estudos prévios.

## CONCLUSÃO

Este estudo permitiu a tradução e adaptação transcultural do instrumento “*Quality of Alimentation*” do inglês para o português do Brasil, deixando-o pronto para as próximas etapas do processo de validação.

## Apêndice 1 “Qualidade da dieta” traduzido e adaptado transculturalmente para o português brasileiro

QUALIDADE DA DIETA

Nome: __________________________________

Sobrenome: __________________________________

Número de meses após a cirurgia: _______ meses.

Como você classificaria sua satisfação geral com seus hábitos alimentares atuais?

**Table:**

Excelente	
Bom	
Aceitável	
Ruim	
Muito ruim	

Por que? __________________________________

Quantas refeições você faz por dia? __________________________________

Qual das seguintes refeições você faz?

**Table:**

Café da manhã	
Almoço	
Jantar	

Qual delas é a sua principal refeição diária? _____________________________

Você come entre as refeições?

**Table:**

Sim	
Não	

Se sim, quando?

**Table:**

Manhã	
Tarde	
Noite	

Você consegue comer todos os tipos de alimentos?

**Table:**

Sim	
Não	

Por favor, avalie o que você acha de comer os seguintes alimentos?

**Table:**

	Fácil	Um pouco difícil	Impossível
Carne vermelha			
Carne branca			
Salada			
Vegetais			
Pão			
Arroz			
Massa			
Peixe			

Existem outros tipos de alimentos que você não consegue comer?

________________________________________________________________

Você vomita/regurgita?

**Table:**

Diariamente	
Frequentemente (mais de duas vezes por semana)	
Raramente	
Nunca
